# Cross-modal integration in the brain is related to phonological awareness only in typical readers, not in those with reading difficulty

**DOI:** 10.3389/fnhum.2013.00388

**Published:** 2013-07-23

**Authors:** Chris McNorgan, Melissa Randazzo-Wagner, James R. Booth

**Affiliations:** Developmental Cognitive Neuroscience Laboratory, Department of Communication Studies and Disorders, Northwestern UniversityEvanston, IL, USA

**Keywords:** dyslexia, functional MRI, audiovisual integration, reading development, developmental disorder, learning disability

## Abstract

Fluent reading requires successfully mapping between visual orthographic and auditory phonological representations and is thus an intrinsically cross-modal process, though reading difficulty has often been characterized as a phonological deficit. However, recent evidence suggests that orthographic information influences phonological processing in typical developing (TD) readers, but that this effect may be blunted in those with reading difficulty (RD), suggesting that the core deficit underlying reading difficulties may be a failure to integrate orthographic and phonological information. Twenty-six (13 TD and 13 RD) children between 8 and 13 years of age participated in a functional magnetic resonance imaging (fMRI) experiment designed to assess the role of phonemic awareness in cross-modal processing. Participants completed a rhyme judgment task for word pairs presented unimodally (auditory only) and cross-modally (auditory followed by visual). For typically developing children, correlations between elision and neural activation were found for the cross-modal but not unimodal task, whereas in children with RD, no correlation was found. The results suggest that elision taps both phonemic awareness and cross-modal integration in typically developing readers, and that these processes are decoupled in children with reading difficulty.

Multisensory, or audiovisual, integration of letters and speech sounds is considered a prerequisite to reading development (Share, 1995). Though processing orthographic or phonological linguistic representations clearly involves a wide cortical network (e.g., attention, semantic processing) a sub-network of cortical regions is strongly associated with processing and integrating orthographic and phonologic representations. This network includes the fusiform gyrus (FG), which is implicated in the processing of orthographic representations (Shaywitz et al., 2002; McCandliss et al., 2003; Dehaene and Cohen, 2011), posterior superior temporal gyrus (pSTG), which is implicated in processing phonologic representations, (Demonet et al., 1992; Booth et al., 2002a) and the posterior superior temporal sulcus (pSTS), which is implicated in audiovisual integration across a wide range of domains (Calvert, 2001; Van Atteveldt et al., 2009; Blau et al., 2010). Because reading entails integrating information from these two representational systems, understanding how cross-modal integration operates in normal and disordered reading may provide insight into the root causes underlying reading difficulty.

Converging evidence from event-related potential (ERP) and functional magnetic resonance imaging (fMRI) studies shows that children with dyslexia demonstrate weaker audiovisual integration of letters and speech sounds, suggesting that reading dysfluency may be partly attributable to difficulties in audiovisual integration. For example, ERP studies of letter-sound integration found that deviant letter-sound pairs produced a mismatch negativity effect in dyslexic readers only given a longer time window, similar to younger reading skill-matched but not age-matched control children, indicating a slow maturational component of audiovisual integration (Froyen et al., 2011). A series of pediatric fMRI studies by Blau et al. (2010) also demonstrated enhanced letter-sound integration in audiovisual conditions for fluent readers compared to dyslexic readers. These studies collectively identified an audiovisual integration network for reading including the planum temporale (PT) in the STG and pSTS in which cross-modal activation differentiated typically-developing and dyslexic children. Taken together, these neurophysiological and imaging studies suggest that children with dyslexia demonstrate reduced audiovisual integration of letters and speech sounds.

Previous studies have explored the mechanisms of audiovisual integration at the level of grapheme to phoneme correspondence, or at a small grain size. Given the inconsistency of the English orthography at the smaller grain sizes, large grain sizes play a greater role in early reading development because they provide greater consistency (Ziegler and Goswami, 2005). Fluent reading in English necessitates processing of larger grain sizes (e.g., words, syllables or rimes) because processing of smaller grain sizes utilizing a letter-by-letter decoding strategy will only be successful with words that have consistent grapheme to phoneme correspondences. Few studies, however, have explored audiovisual integration for whole word reading. Snowling (1980) compared dyslexic and reading-age matched readers' nonword recognition ability in unimodal (auditory only and visual only) and cross-modal (auditory-visual, visual-auditory) conditions, and noted the greatest difference in discrimination sensitivity between the two groups was in the visual-auditory condition. In the neuroimaging domain, Kast et al. (2011) compared fMRI activations of dyslexic and non-dyslexic adults during a lexical decision task presented in unimodal (auditory only and visual only), and cross-modal (auditory-visual) conditions. They found that the dyslexic group showed altered brain activation for the cross-modal condition in right STS and left supramarginal gyrus, both of which are implicated in cross-modal conversion (Calvert et al., 2000; Booth et al., 2002b). We stress, however, that Kast et al. assessed overall group differences in adult readers. Though potentially diagnostically useful for an older population, their study does not address whether these differences are present during early childhood and thus potentially impact the acquisition of reading skill, nor relate these differences to an independent cognitive measure. Thus, there is some evidence to suggest that impaired audiovisual integration at larger grain sizes underlies the reading difficulties experienced by dyslexic individuals, though the mechanism through which these impairments influence the acquisition of reading skill remains unclear.

As suggested earlier, however, audiovisual integration is a complex process, as it involves two very different representational systems. Thus, failure to properly integrate phonological and orthographic representations could be attributable to a failure of the phonological, orthographic or integration processes in isolation or in combination. A large body of behavioral and neuroimaging literature argues that reading fluency depends critically on phonological awareness skills. For example, dyslexic adults, relative to controls, display reduced phonological awareness, despite having intact phonological representations, and this reduced awareness is predictive of deficits in two measures of phonological decoding: nonword reading and nonsense passage reading (Mundy and Carroll, 2012). Phonological awareness skills have been shown to predict reading success in several alphabetic languages (Ziegler and Goswami, 2005). These skills appear to develop hierarchically from larger units at the word/syllable level to an intermediate rime/onset level, and ultimately to the smallest, phonemic level (Anthony and Francis, 2005).

Some have argued that phonemic-level awareness is a result of increased sensitivity to phonemes by exposure to orthography (Ziegler and Goswami, 2005), consistent with the argument that this skill is an experience-based developmental consequence of reading in typically developing readers—a position supported by numerous studies showing the influence of orthography on phonological processing (Stuart, 1990; Castles et al., 2003; Desroches et al., 2010). Several behavioral studies have shown that orthographic knowledge impinges on phoneme judgments. For example, given words like *pitch* and *rich* with the same number of sounds but not letters, typically developing readers perceived *pitch* as having a greater number of phonemes (Ehri and Wilce, 1980). This influence of orthography on phoneme judgment has been shown to emerge in preschool-aged children (Castles et al., 2011), suggesting that this cross-modal influence may accompany learning of the alphabetic principle, but continue as a child learns to read.

Phonological awareness tasks involving manipulation of smaller grain sizes (e.g., more reliant on knowledge of the alphabetic principle) have been more specifically referred to in the literature as phonemic awareness tasks. A recent meta-analysis showed that early phonemic awareness is closely related to growth of word reading and is more highly correlated with reading skill than both rime-level awareness and verbal short-term memory (Melby-Lervåg et al., 2012). Elision is a phonemic awareness task, typically measured in English by standardized assessment in which increasingly smaller segments must be removed from the stimulus at increasingly higher levels of linguistic complexity, from words down to phonemes within clusters (Wagner et al., 1999). In this task, participants repeat a verbally presented word (e.g., *CUP*, or /kәp/) and then verbally produce a novel word after a particular phoneme has been deleted (e.g., /kәp/ without the /k/ sound produces /әp/, or *UP*) Elision has been noted as a sensitive measure of phonological skill that discriminates between high and low ability readers better than rhyming and phoneme identification (Vloedgraven and Verhoeven, 2007).

Because elision places large demands on phonemic awareness, it may tap into the processes that are critical for orthographic-phonologic integration, and thus predict cross-modal integration performance. A cross-modal influence of orthographic knowledge on a phonemic awareness task would support the view that phonemic awareness is a byproduct of increased reading ability (Ziegler and Goswami, 2005). Several behavioral studies showing orthographic knowledge impinges on phoneme judgments support this position. Take for example the word *BIND*(/bajnd/), when instructed to omit the /n/ phoneme. The deletion of /n/ produces BIDE (/bajd/). Though irrelevant to the task, when the grapheme corresponding to /n/ is deleted from BIND, the product is BID (/bid/). Stuart (1990) showed that children often produced the result of the grapheme, rather than phoneme deletion (e.g., producing /bid/), suggesting they enlisted orthographic knowledge during the task. Another study using an elision task involving an orthographic transparency manipulation with transparent words in which the sound to be deleted had a one-to-one phoneme grapheme correspondence (delete /f/ from *rafter*), and opaque words, in which the sound to be deleted was a silent letter or a biphonemic grapheme (delete /n/ from *knuckle*). Results indicated that children found it more difficult to delete phonemes from opaque items, indicating orthographic influence on phonemic awareness (Castles et al., 2003). Collectively, these results support the notion that orthographic knowledge changes phonological awareness at the phonemic level.

Despite the strong ties between elision and audiovisual integration during reading, only one neuroimaging study to date has examined the relationship between elision and modality-related performance. Frost et al. (2009) examined whether elision skill was correlated with functional activation for unimodal (print vs. speech) tasks in typically developing children. They found correlations between elision and activity in left superior temporal cortex close to the PT and STS and in left occipitotemporal regions including the FG. In the left superior temporal cortex, higher phonemic awareness skill was associated with greater activation when processing print, equivalent to when processing speech. In left occipitotemporal cortex, higher phonemic awareness skill was correlated with less activation when processing speech. These results suggest that higher elision skill is associated with greater specialization of the orthography-phonology sub-network for print, but that the effects of increased audiovisual integration are most pronounced in phonological regions when processing print representations. This is consistent with the finding that elision is positively correlated with print-related activations in left STG, left FG, and left thalamus (Pugh et al., 2013). In summary, although elision is considered a measure of phonemic skill, it seems to be influenced by orthographic knowledge in developing readers, and therefore, may be sensitive to the audiovisual nature of literacy acquisition.

Because previous studies have not examined the role of phonemic awareness (i.e., elision) in unimodal vs. cross-modal tasks, there is no direct evidence relating this skill to audiovisual integration. Moreover, though elision skill has been shown to be diagnostic of reading difficulty, it remains unclear whether the specificity and sensitivity of this measure is a result of it tapping into processes underlying audiovisual integration. Finally, previous audiovisual studies have examined letter-speech congruency, so it is not known whether developmental or disability differences in audiovisual integration apply to larger grain sizes, despite these grain sizes being fundamental to English. To address these issues, the current study examined unimodal (auditory only) and cross-modal (auditory-visual) processing of words in typically developing (TD) readers and those with reading difficulty (RD). We focused our analyses to three regions in a left hemisphere sub-network implicated in orthographic (FG) and phonolologic (PT) processing, and audiovisual integration (pSTS), consistent with a model of audiovisual integration in reading (Van Atteveldt et al., 2009; Blau et al., 2010). In this model, the pSTS is believed to have reciprocal interconnections with the PT and FG, permitting top-down influence of orthography on phonological processing in PT and the converse top-down influence on orthographic processing in the FG.

Stimulus congruency (i.e., whether two items match along a critical dimension) is often used in the investigation of cross-modal interaction, as it demonstrates that the processing of one item influences the processing of the other. Following other studies investigating reading-related cross-modal development (e.g., Froyen et al., 2008; McNorgan et al., 2013), we assessed the neural response to inter-stimulus congruency. Our question concerned whether elision is primarily sensitive to phonological awareness (manipulation of sounds in spoken language only) or is sensitive to access of orthography from spoken words. Consequently, it was most appropriate to assess these congruency effects under conditions in which participants are presented spoken words only (unimodal auditory) and requiring audiovisual integration (cross-modal). Because of the central role that it is assumed to play in audiovisual integration, we hypothesized that elision would be positively correlated with pSTS activity in the cross-modal condition. Because it should directly influence both phonological and orthographic processing areas, skill-dependent audiovisual integration effects were additionally hypothesized for the FG and PT suggesting interaction between neural systems involved in processing speech and print. Finally, we investigated whether a differential relationship of phonemic skill with audiovisual integration would be present in TD compared RD children.

## Materials and methods

### Participants

A group of 13 typically developing (TD) (7 males; mean age = 11 years, 0 months; range = 8 years, 0 months to 13 years, 7 months) and 13 children with reading difficulty (RD) (7 males; mean age = 11 years, 0 months; range = 9 years, 5 months to 12 years, 6 months) participated in the present study. All participants were native English speakers, right handed, had normal or corrected-to-normal vision, and had no history of psychiatric illness, neurological disease, learning disability or attention deficit hyperactivity disorder (ADHD). Participants were recruited from the Chicago metropolitan area. Informed consent was obtained from participants and their parents, and all procedures were approved by the Institutional Review Board at Northwestern University.

Prior to admission to the study, we evaluated children's non-verbal IQ using the Wechsler Abbreviated Scale of Intelligence and reading-related skill using the Word Identification, Word Attack and Reading Fluency subtests of the Woodcock Johnson Tests of Achievement—III (WJ III) and the Sight Word Efficiency and Phonetic Decoding Efficiency subtests of the Test of Word Reading Efficiency (TOWRE). Participants in the TD group had no subtest standardized score less than 95, and an average across the 5 reading subtests exceeding 100. Participants in the RD group had to have at least one subtest standardized score less than or equal to 85 and an average across the 5 reading subtests of less than 100. Other demographic and non-reading variables were matched as closely as possible. The minimum performance IQ cutoff for participants in both groups was 79 in all performance IQ subtests, and experimental task performance for all participants had to be better than chance for all experimental conditions of interest. Group mean and standard deviations of scaled scores across these standardized measures for the TD and RD participants are presented in Table 1, which shows that the TD and RD groups significantly differed across all standardized measures of reading skill, but not for performance (i.e., non-verbal) IQ.

**Table 1:** **Mean scaled scores and standard deviations (in parentheses) for standardized tests of achievement for typically developing (TD) and reading difficulty (RD) groups**.

**Standardized measure**	***TD***	***RD***	***t*_(24)_**	***p***
Word identification (WJ III)	116.4 (13.9)	89.6 (8.0)	6.03	<0.001
Word attack (WJ III)	115.0 (9.7)	92.2 (6.1)	7.19	<0.001
Reading fluency (WJ III)	115.4 (9.8)	87.7 (11.6)	6.55	<0.001
Sight word efficiency (TOWRE)	115.9 (10.7)	87.1 (14.4)	5.81	<0.001
Phonetic decoding efficiency (TOWRE)	120.2 (14.9)	85.3 (9.7)	7.06	<0.001
Mean of 5 reading subtests	116.6 (9.7)	88.4 (7.4)	8.36	<0.001
Performance IQ (WASI)	125.5 (11.6)	107.0 (17.1)	1.35	0.19

*t_(24)_, t-score; p, independent-samples t-test significance with 24 degrees of freedom; WJ-III, Woodcock Johnson Tests of Achievement—III; TOWRE, Test of Word Reading Efficiency; WASI, Wechsler Abbreviated Intelligence Scale*.

We measured each participant's phonemic awareness using the elision subtest of the Comprehensive Test of Phonological Processing (CTOPP). Briefly, in this task, participants are instructed to repeat a verbally presented word (e.g., “Say /tajgәr/”) and then instructed to verbally produce the word with the specified phoneme omitted (e.g., “Now say /tajgәr/ without the /g/ sound”), wherein the product of the elision is a valid English word (e.g., removing the /g/ from TIGER -/tajgәr/ produces TIRE -/tajәr/). Elision scores reflect the number of correct elision transformations on a set of 20 progressively difficult target items.

### Experimental procedure

#### Rhyme judgment task

**Figure 1:**
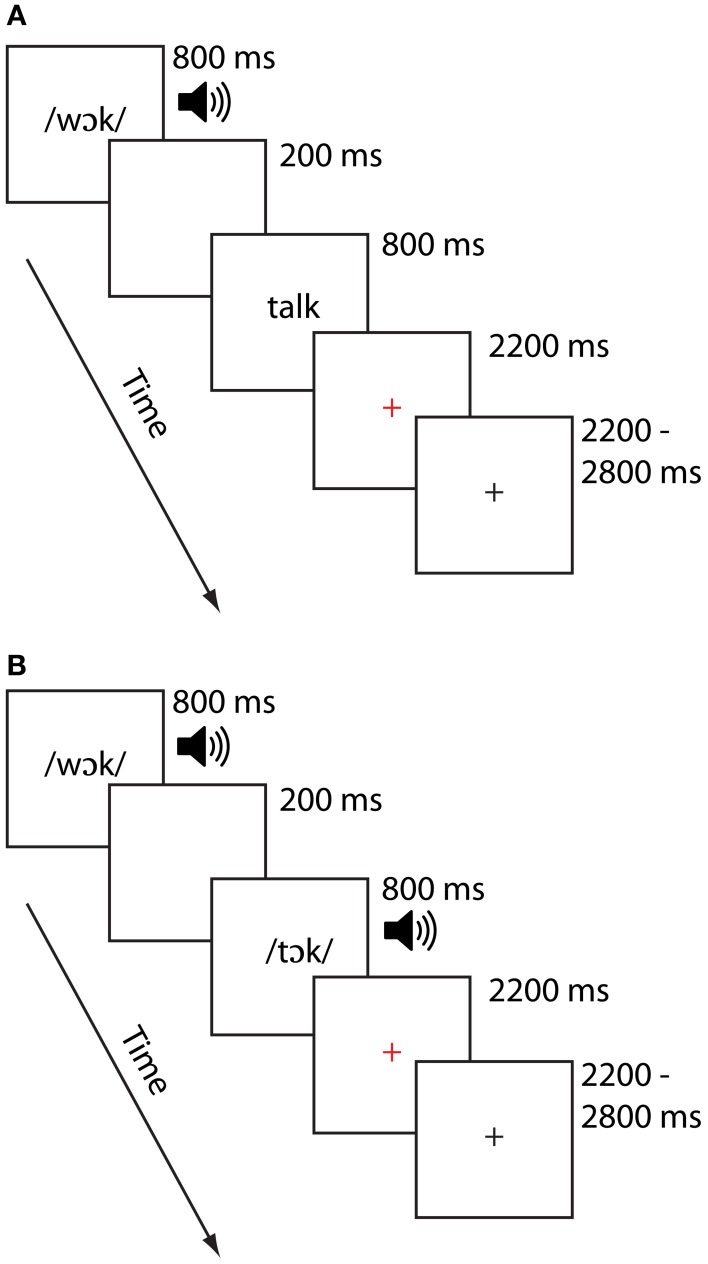
**Task diagram for the AV Cross-modal task (A) and AA Unimodal task (B)**.

On each trial, participants were presented with paired stimuli the order of which was counterbalanced across participants. For each scanning session, stimuli were presented in one of two modality conditions: In the cross-modal auditory/visual (AV) condition, the first item was presented auditorily and the second was presented visually. In the unimodal auditory/auditory (AA) condition, both items were presented in the auditory modality. Previous investigations of cross-modal lexical processing research (e.g., Van Atteveldt et al., 2004; Froyen et al., 2008) similarly employed auditory-then-visual presentations, motivating the task design for that modality condition. Half the pairs of stimuli rhymed and half did not, and participants were asked to make a rhyme judgment response by pressing one of two keys on a handheld keypad. Participants were asked to respond as quickly and as accurately as possible, using their right index finger for a yes (rhyme) response and their right middle finger for a no (non-rhyme) response. Participants participated in two runs for each modality condition, each lasting approximately 7 min. Participants generally saw the AV condition followed by the AA condition, though this varied across participants as factors such as task accuracy and movement necessitated reacquiring data. An independent samples *t*-test on the time interval between the AV and AA tasks confirmed, however failed to show a difference between these time intervals, *t*_(24)_ = 1.24, *p* > 0.23. Thus, the two groups did not systematically differ with respect to the order in which they performed the tasks. Each stimulus item was presented for 800 ms, separated by a 200 ms interstimulus interval. Participants were free to respond as soon as the second stimulus item was presented. A red cross appeared for 2200 ms following the presentation of the second word, signaling to the participant to respond if they had not already done so. Responses made after the red cross disappeared from the screen were not recorded and counted as errors. A jittered response interval duration of between 2200 and 2800 ms was used to allow for deconvolution of the signal associated with each condition. The sequence and timings of lexical trial events are illustrated for each modality in Figure 1. Stimulus pairs varied in terms of their orthographic and phonological similarity, and were presented in one of four similarity conditions (24 pairs per condition). There were two phonologically similar (i.e., rhyming) conditions, one with orthographically similar pairs (O+P+; e.g., CAGE-RAGE) and another with orthographically dissimilar pairs (O−P+; e.g., GRADE-LAID). There were also two phonologically dissimilar (i.e., nonrhyming) conditions, one with orthographically similar pairs (O+P−; e.g., SMART-WART) and one with orthographically dissimilar pairs (O−P−; e.g., TRIAL-FALL). All words were monosyllabic, having neither homophones nor homographs, and were matched across conditions for written word frequency in children (Zeno, 1995) and the sum of their written bigram frequency (English Lexicon Project, http://elexicon.wustl.edu). We restricted our analyses to the two rhyming conditions (i.e., those associated with “yes” responses) to avoid introducing response-related confounds related to making “yes” vs. “no” judgments. Fixation trials (24 for each run) were included as a baseline and required the participant to press the “yes” button when a fixation-cross at the center of the screen turned from red to blue. Perceptual trials (12 trials for each run) were included for a related study (McNorgan et al., 2013). Perceptual trials comprised two sequences containing tones (AA), or tones followed by glyphs (AV). These stimuli were presented as increasing, decreasing or steady in pitch (for auditory stimuli) or height (for visual stimuli). Participants were required to determine whether the sequences matched (e.g., two rising sequences) or mismatched (e.g., a falling sequence followed by a steady sequence) by pressing the “yes” button to indicate a match, and the “no” button otherwise. The timing for the fixation and perceptual trials were the same as for the lexical trials.

#### Functional MRI data acquisition

Participants were positioned in the MRI scanner with their head secured using foam pads. An optical response box was placed in the participant's right hand to log responses. Visual stimuli were projected onto a screen, which participants viewed via a mirror attached to the inside of the head coil. Participants wore sound attenuating headphones to minimize the effects of the ambient scanner noise. Images were acquired using a 3.0 Tesla Siemens Trio scanner. The BOLD (blood oxygen level dependent) signal was measured using a susceptibility weighted single-shot EPI (echo planar imaging) method. Functional images were interleaved from bottom to top in a whole brain acquisition. The following parameters were used: TE = 20 ms, flip angle = 80°, matrix size = 128 × 120, field of view = 220 × 206.25 mm, slice thickness = 3 mm (0.48 mm gap), number of slices = 32, TR = 2000 ms, voxel size = 1.72 mm × 1.72 mm. Before functional image acquisition, a high resolution T1-weighted 3D structural image was acquired for each subject (TR = 1570 ms, TE = 3.36 ms, matrix size = 256 × 256, field of view = 240 mm, slice thickness = 1 mm, number of slices = 160, voxel size = 1 mm × 1 mm).

#### Functional MRI data preprocessing

fMRI data were analyzed using SPM8 (Statistical Parametric Mapping, http://www.fil.ion.ac.uk/spm). ArtRepair software (http://cibsr.stanford.edu/tools/human-brain-project/artrepair-software.html) was used during image preprocessing to correct for participant movement. Images were realigned in ArtRepair, which identified and replaced outlier volumes, associated with excessive movement or spikes in the global signal, using interpolated values from the two adjacent non-outlier scans. Outlier scans were defined as those for which a signal change of more than 1.5% from the mean or movement of 4 mm or more along any axis was detected. No more than 10% of the volumes from each run and no more than 4 consecutive volumes were interpolated in this way. For each participant, a single attempt was made to reacquire runs requiring replacement of more than 10% of the volumes or more than 4 consecutive volumes. Slice timing was applied to minimize timing-errors between slices. Functional images were co-registered with the anatomical image, and normalized to the Montreal Neurological Institute (MNI) ICBM152 T1 template. This template is well-defined with respect to a number of brain atlas tools and the MNI coordinate system, and stereotactic space for children within the age range included in our study has been shown to be comparable to that of adults (Burgund et al., 2002; Kang et al., 2003). Images were smoothed using a 2 × 2 × 4 non-isotropic Gaussian kernel.

## Behavioral analyses

We restricted our analyses to the rhyming conditions (i.e., those with a “yes” response), and thus within the context of our experiment, congruency referred to whether the spelling of rhyming pairs matched (i.e., congruent or O+P+, as in CAGE-RAGE) or mismatched (i.e., incongruent or O−P+, as in GRADE-LAID). The congruency effect was thus a measure of the difference between responses, whether in terms of behavior or brain activity, between these two conditions. Because stimulus pair congruency was assumed to influence behavioral performance and BOLD activity for the task (Bitan et al., 2007), a 2 group × 2 task modality analysis of variance (ANOVA) was conducted on the congruency effect (i.e., the difference between congruent and incongruent conditions) to parallel the fMRI congruency effect analysis, with modality as a within-subjects independent variable and group as a between-subjects variable. The dependent variables were the congruency effects for accuracy rates and decision latencies of correct responses.

### fMRI analyses

Statistical analyses were calculated at the first-level using an event-related design with all four lexical conditions (O+P+, O−P+, O−P−, O+P−), the fixation condition, and the perceptual condition included as conditions of interest. Interpolated volumes were deweighted, and the first 6 volumes of each run, during which a fixation cross was presented, were dropped from the analyses. A high pass filter with a cut off of 128 s was applied. Lexical, fixation and perceptual pairs were treated as individual events for analysis and modeled using a canonical hemodynamic response function (HRF). Voxel-wise *t*-statistic maps were generated for each participant contrasting the balanced rhyme (O+P+, O−P+) vs. fixation (rhyme—fixation) and congruent vs. incongruent rhyme (O+P+ —O−P+) within each modality condition (6 contrasts). Group-level results were obtained using random-effects analyses by combining subject-specific summary statistics across the group as implemented in SPM8. We were primarily interested in the relationship between elision skill and cross-modal integration in TD and RD children, rather than absolute differences between groups or task modality. Thus, these maps were calculated for the purpose of identifying voxels that were reliably activated for the lexical task for constraining our region of interest definitions (see below) and were not analyzed further.

### Region of interest definitions

We focused on the neural responses to orthographic congruency in the PT, FG and pSTS—three anatomical regions associated with phonological, orthographic and cross-modal processing, respectively. Because it was plausible that RD participants would show weaker overall BOLD responses, we defined these regions anatomically and functionally in two steps. This procedure ensured that group differences could not be attributed to a comparison between robust vs. noisy data. In the first step, an atlas-based anatomical definition of left PT was taken from the Harvard-Oxford Cortical Structure Atlas. This atlas is probabilistic, meaning that one or more anatomical labels are assigned to each voxel with an associated probability reflecting the likelihood that the voxel is found in that anatomical region. We selected those voxels for which the PT was the most probable label. That is, if a voxel had been assigned the PT label (with any probability), and if that the probability for belonging to the PT was greater than the probability associated with any other single region, that voxel was included in the PT definition. An atlas-based definition of left FG was taken from the automated anatomic labeling (AAL) atlas included with SPM 8. An atlas-based definition of pSTS was created by intersecting the AAL definitions of left superior temporal gyrus and middle temporal gyrus, each dilated by 4 mm along each axis. The overlapping region defines the sulcus because it follows the line that delineates these immediately adjacent atlas definitions. Posterior STS was defined as those voxels posterior to y = −40, or roughly the posterior third of the volume. The use of two anatomical atlases was necessitated by the fact that not all regions were defined in a single atlas. STS is not defined in either the AAL or Harvard-Oxford probabilistic atlases, however it was relatively straightforward to define STS as described above using the WFU PickAtlas SPM toolbox (http://fmri.wfubmc.edu/), which interfaces with the AAL atlas. Unfortunately, the AAL atlas does not include a definition for PT, necessitating the use of the Harvard-Oxford atlas. Finally, though FG is defined in both atlases, there was no a priori justification to choose one atlas over the other, and so we selected the AAL template FG definition to provide consistency between this and another related study for which the FG definition had been previously from the AAL atlas.

**Figure 2:**
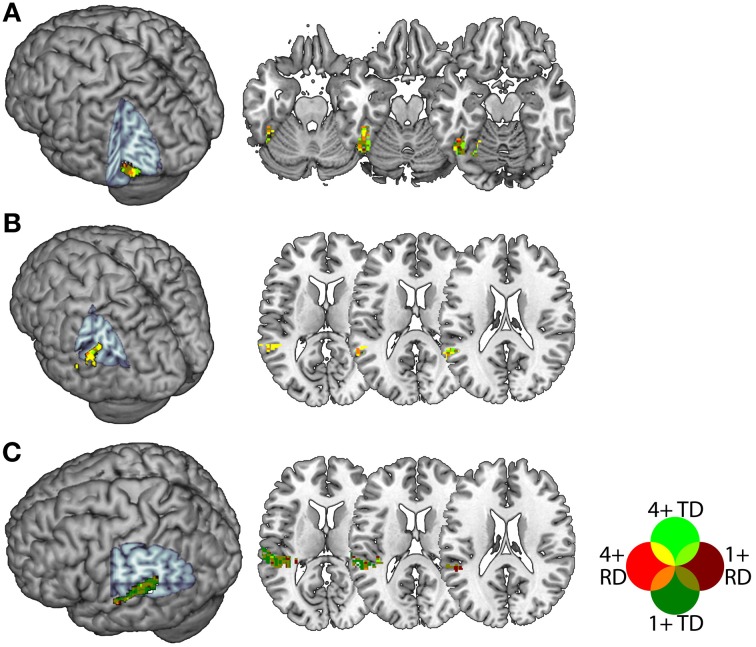
**Voxels included in the Fusiform Gyrus (A), posterior Superior Temporal Sulcus (B) and Planum Temporale (C) ROIs.** Voxels appearing in TD ROIs are green and those appearing in RD ROIs are red, with brighter colors indicating voxels appearing in more (4+ of 13) participants. Voxels appearing in ROIs for both groups appear in yellow, orange, olive or bright green.

In a second step, we intersected the atlas-based definitions with statistically thresholded contrast maps in order to constrain our analyses to voxels that were sensitive to congruency for both groups. This was because purely atlas-based ROIs might plausibly be biased even if overall group differences in congruency-related activity were below statistical threshold. Within each of these atlas-based definitions, we selected for each individual the 30 voxels with the highest positive *t*-statistic in the congruent vs. incongruent first-level contrast for each of the AA and AV tasks, thresholded at a liberal alpha of 0.1 (uncorrected). This threshold was a compromise between the need to select a sufficient number of voxels for both TD and RD participants, and the need to ensure that ROIs contained voxels that were reasonably sensitive to congruency, and 30 voxels was the largest multiple of 5 for which an ROI could be created for all participants, regions and tasks. This procedure thereby selected voxels demonstrating a congruency effect in both task modalities and in both groups, and produced ROIs of comparable extent across individuals. Note that because we selected the top thirty voxels in each individual's *t*-statistic map, there was no common threshold across participants (i.e., the 30th highest *t*-statistic in each map varied by individual), apart from reaching the minimum uncorrected statistical threshold of 0.1. Figure 2 depicts the voxels included in each ROI collapsed across participants within each group. For pSTS and FG, a large proportion of voxels were common to many participants from both groups, whereas within PT, there were more individual differences with respect to the voxels showing a congruency effect in both task modalities. The ROIs contained an average of 56, 51 and 58 voxels for the FG, pSTS and PT, respectively, and none had fewer than 44 voxels.

The preceding two steps served to create the ROI definitions. Congruency effects were calculated within each participant by finding the difference between the mean signal among voxels in each ROI for the congruent vs. incongruent rhyming conditions. The congruency effect was calculated separately for the AA and the AV task modalities. We calculated the Pearson correlation between these congruency effects and elision for each group to assess whether elision skill was related to the sensitivity of the BOLD response to inter-item congruency for TD and RD participants, and used the Fischer *Z* test to directly compare the TD and RD correlations.

### Results

#### Behavioral analysis

Behavioral task performance is presented in Table 2. No overall difference was observed between the accuracy congruency effect (i.e., the difference between O+P+ and O−P+ accuracy) for the AV (*M* = −0.05, *SE* = 0.03) and AA (*M* = −0.06,*SE*= 0.02) tasks, *F*_(1, 24)_ = 0.01,*p* > 0.90. The TD (*M* = −0.04, *SE* = 0.02) and RD accuracy congruency effects (*M*= −0.03, *SE* = 0.02) were equivalent, *F*_(1,24)_ = 0.01, *p* > 0.90, and there was no group by task modality interaction, *F*_(1,24)_ = 0.16, *p* > 0.60.

**Table 2:** **Mean accuracy and decision latency by task modality and congruency condition (standard deviations in parentheses) for TD and RD participants**.

		**Accuracy**		**Decision latency**	
		**AV**	**AA**	**AV**	**AA**
TD	Congruent	0.80 (0.16)	0.81 (0.13)	1237 (286)	1492 (284)
	Incongruent	0.84 (0.10)	0.87 (0.09)	1176 (372)	1440 (250)
RD	Congruent	0.68 (0.20)	0.71 (0.19)	1583 (337)	1702 (297)
	Incongruent	0.74 (0.19)	0.76 (0.18)	1490 (279)	1690 (283)

Decision Latency indicated in milliseconds.

There was similarly no overall difference between decision latency congruency effects for the AV (*M* = 33 ms,*SE* = 0.29 ms) and AA (*M* = 32 ms, *SE* = 20 ms) tasks, *F*_(1,24)_ = 0.00, *p* > 0.90. The TD (*M* = 42 ms, *SE* = 20 ms) and RD decision latency congruency effects (*M* = 24 ms, *SE* = 20 ms) were equivalent, *F*_(1,24)_ = 0.43, *p* >0.50, and there was no group by task modality interaction, *F*_(1, 24)_ = 0.27, *p* > 0.60.

Thus, though the TD participants clearly outperformed the RD participants in terms of both accuracy and decision latency, the behavioral congruency effects were equivalent across the modality conditions and between the TD and RD groups.

### Region of interest analysis

**Figure 3:**
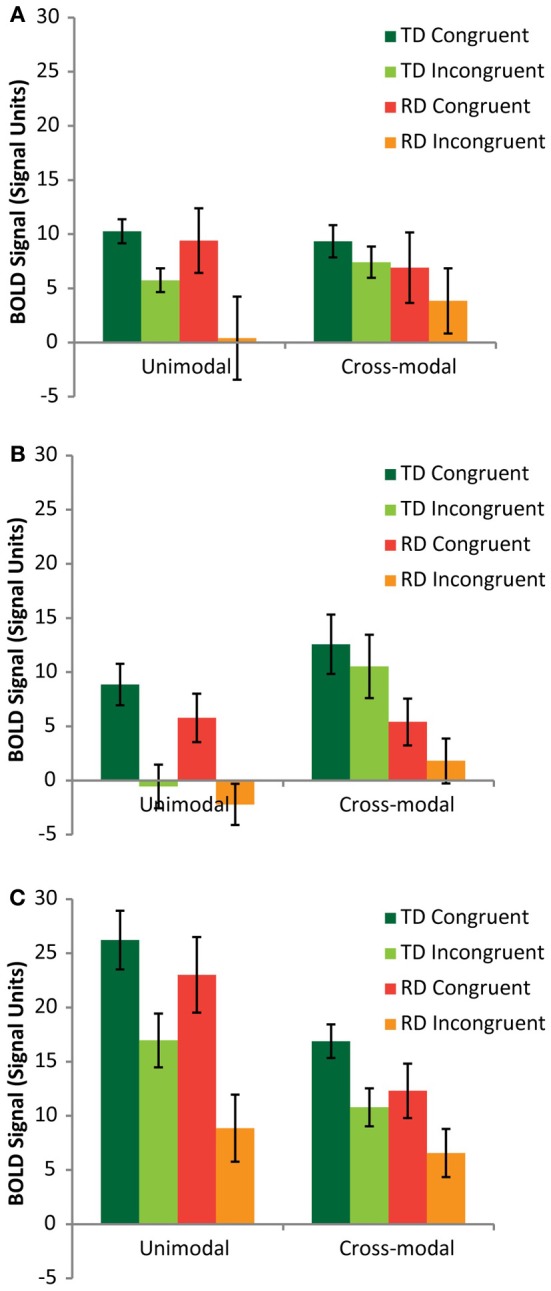
**Mean rhyming condition vs. fixation signal graphed for TD (green/lime) and RD (red/orange) participants from the Fusiform Gyrus (A), posterior Superior Temporal Sulcus (B) and Planum Temporale (C) ROIs**.

A mixed model analysis of variance (ANOVA) was carried out on the neural congruency effects calculated for each condition and each ROI, using region (FG vs. pSTS vs. PT) and modality (AA vs. AV) as within-subjects variables and group (TD vs. RD) as a between-subjects variable. There was a significant main effect of region [*F*_(2,48)_ = 9.14, *p* < 0.001], driven by a significantly greater congruency effect in the PT (*M* = 8.81, *SE* = 0.86) compared to FG (*M* = 5.75, *SE* = 1.20) and pSTS (*M* = 4.63, *SE* = 0.97). The congruency effect was also greater for the AA condition (*M* = 9.06, *SE* = 1.01) compared to the AV condition (*M* = 3.74, *SE* = 1.11). Figure 3 presents the BOLD signal for each rhyming condition compared to baseline for each task modality and each ROI to aid in interpreting these results. Both groups showed similar relationships between congruent and incongruent signal for both modalities, with the RD group tending to exhibit weaker signal overall, but also showing greater variance. These main effects should be interpreted with caution, however for two important reasons. First, there was additionally a three-way interaction between region, mode and group [*F*_(2,24)_ = 3.39, *p* = 0.04]. Second, and more importantly, as the remaining analyses show, and as we hypothesized, these congruency effects had a group and regional dependency on elision skill.

**Figure 4:**
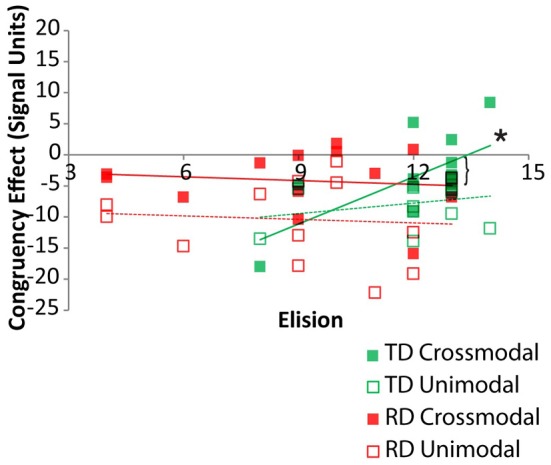
**Congruency effects for TD (green) and RD (red) participants as a function of Elision score in the cross-modal (solid) and unimodal (dashed/outlined) task conditions.** Significant correlations are indicated by an asterisk. Significant Fischer's Z test of differences between correlation coefficients is indicated by a bracket.

Pearson correlations between elision performance and the neural congruency effect within each ROI were calculated over the set of ROIs for the two groups. Across all ROIs, there was a significant correlation between elision and the neural congruency effect for the cross-modal task condition for the TD group, *r*_(11)_ = 0.68, *p* = 0.005, but not for the RD group, *r*_(11)_ = −0.12, *p* = 0.69, and the correlations differed significantly between the two groups, *Fischer* Z = 2.12, *p* = 0.03. Though none of the TD participants were statistical outliers with respect to Elision, we calculated the Spearman correlation between the neural congruency effect and the rank-order transformation of the Elision scores to ensure that the effects were not primarily driven by the two lowest-scoring TD participants. The results were similar, *r*_*s*_(11) = 0.60, *p* = 0.015. The neural congruency effect for the unimodal task within this network was not significantly correlated with elision score for either the TD group, *r*_(11)_ = 0.28, *p* = 0.35, or the RD group, *r*_(11)_ = −0.08, *p* = 0.80, and these correlations did not significantly differ, *Fischer Z* = 0.82, *p* = 0.41. Thus, elision predicts the sensitivity of this sub-network to spelling-sound congruency, but only for typically developing readers and only when engaged in a task requiring the integration of cross-modal information (Figure 4). A follow-up analysis showed the BOLD response to cross-modal congruency was not correlated with any of the standardized measures used as selection criteria, nor was elision skill significantly correlated with any of these measures. Thus, the neural response to cross-modal congruency was tied only to elision which was itself relatively independent of the reading skill measures we used to discriminate TD and RD participants.

**Figure 5:**
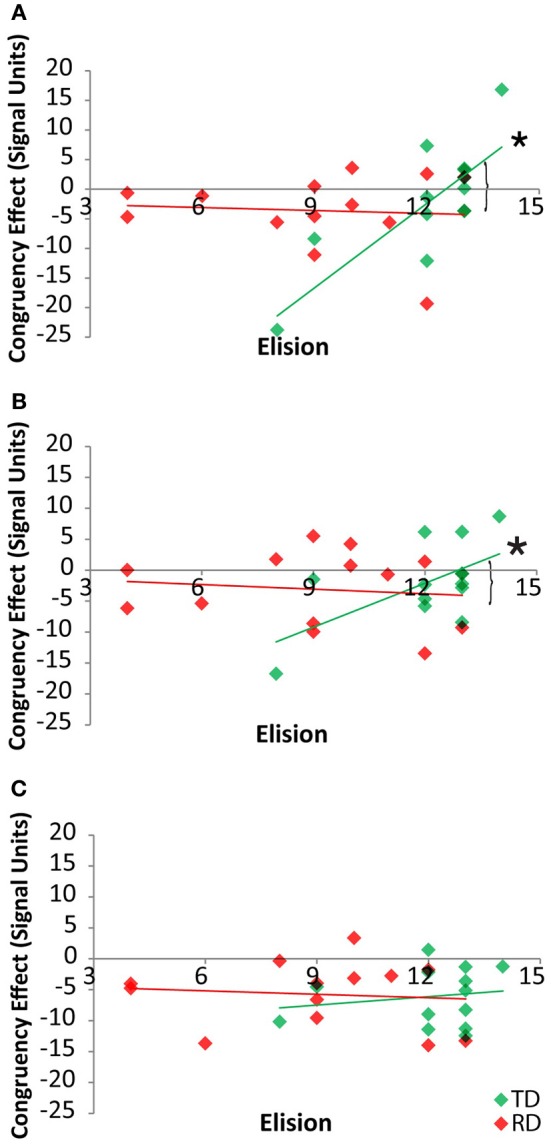
**Correlations between cross-modal congruency effects and Elision score within Fusiform Gyrus (A), posterior Superior Temporal Sulcus (B) and Planum Temporale (C) for TD (green) and RD (red) participants.** Significant correlations are indicated with an asterisk. Significant Fischer's Z test of differences between correlation coefficients are indicated by brackets.

By-region analyses of the neural congruency effect for the cross-modal task condition showed that there was a significant correlation between the congruency effect in FG for the TD group, *r*_(11)_ = 0.80, *p* = 0.001, but not for the RD group, *r*_(11)_ = −0.08, *p* = 0.80, and that these two correlations differed, Fishers Z = 2.64, *p* = 0.004 (Figure 5A). Within the pSTS, there was a significant correlation between the congruency effect and Elision in FG for the TD group, *r*_(11)_ = 0.60, *p* = 0.03, but not for the RD group, *r*_(11)_ = −0.12, *p* = 0.70, and these two correlations differed, *Fishers Z* = 1.82, *p* < 0.03 (Figure 5B). Within the PT, the congruency effect was not correlated with Elision for either the TD group, *r*_(11)_ = 0.17, *p* = 0.57, or the RD group, *r*_(11)_ = −0.1, *p* = 0.75 (Figure 5C) and these correlations did not differ, *Fishers Z* = 0.82, *p* > 0.20. Thus, the pattern of correlations seen across the network between elision and the neural congruency effect appear to be driven by a significant relationship in the FG and pSTS for TD participants in the cross-modal task.

**Figure 6:**
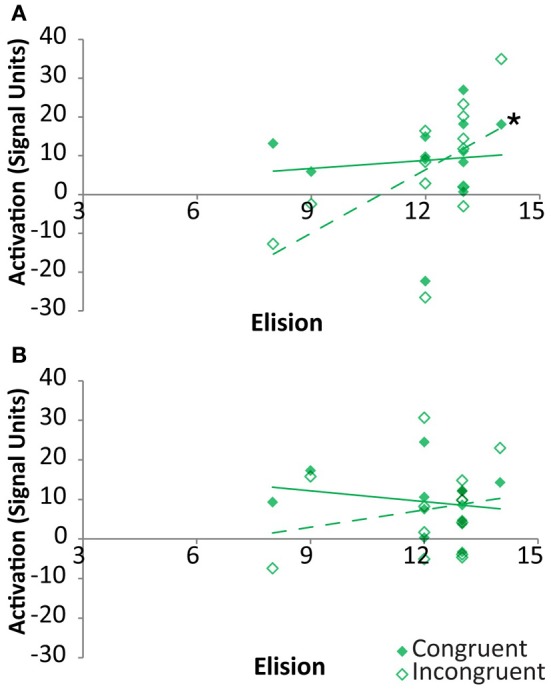
**Correlations between activation and Elision score within TD participants for congruent (solid) and incongruent (outlined) rhyming pairs in the cross-modal task within Fusiform Gyrus (A) and posterior Superior Temporal Sulcus (B).** Significant correlations are indicated with an asterisk.

As indicated above, the neural congruency effect was calculated as the difference between signal for congruent vs. incongruent rhyming items. Thus, it was unclear whether these correlations were primarily driven by either congruency condition in the two regions showing a clear relationship between congruency effect and elision. Within the FG, the signal strength associated with congruent items was not significantly correlated with elision, *r*_(11)_ = 0.1, *p* = 0.75, whereas that associated with incongruent items was strongly positively correlated with elision, *r*_(11)_ = 0.57, *p* = 0.04, though the correlation coefficients did not significantly differ (Figure 6A). Within pSTS, correlations between activation and elision was neither significant for congruent items, *r*_(11)_ = −0.21, *p* = 0.49, nor for incongruent items, *r*_(11)_ = 0.21, *p* = 0.49 (Figure 6B).

## Discussion

This study investigated whether children with reading difficulty (RD) had altered audiovisual integration effects as compared to typically developing (TD) children. TD, but not RD, children showed significant correlations between a measure of phonemic awareness (i.e., elision) and the congruency effect for the cross-modal task. The cross-modal task involved an auditory followed by a visual word that was either orthographically congruent (e.g., lake-cake) or incongruent (e.g., grade-laid). Moreover, there was no significant correlation for elision and the unimodal congruency effect for either group, indicating that elision predicts sensitivity to orthographic congruency, but only for cross-modal processing in the TD children. This pattern suggests two things: First, that elision and cross-modal processing skill are tightly bound in typically-developing readers; and second, that a breakdown in this relationship is associated with difficulties in reading. We note that, at first glance, our failure to find a correlation between elision and activity in the auditory-only condition may appear counter-intuitive, given both the presumed reliance of elision on phonemic awareness, and the presumed reliance of our rhyming judgment task on phonological processing. This pattern, however, reinforces the argument that elision is particularly sensitive to the presence of orthographic input in TD children. That is, in the auditory-only condition, orthographic conflict in the O- conditions is not relevant to the task. Though this conflict may influence phonological and automatic orthographic processing of auditorily-presented stimuli, elision skill does not predict how it influences performance. Rhyming judgments appear to be made largely on the basis of phonological similarity between stimuli in this task. For the cross-modal condition, however, elision predicts the degree to which orthographic conflict influences the network, and consequently produces a cross-modal congruency effect.

Correlations across the network between elision and cross-modal congruency were driven by a significant relationship in fusiform gyrus (FG) and posterior superior temporal sulcus (pSTS) for the TD children. Though there is little disagreement that, as part of the visual processing stream, FG is involved in orthographic processing, evidence for a role of FG in cross-modal processing is ambiguous, with some studies indicating that the region is predominantly specialized for unimodal orthographic processing (e.g., Booth et al., 2002b; Blau et al., 2010), and others suggesting that function of the region is dynamically determined by interactions with other areas involved language processing (e.g., Price et al., 2003; Price and Devlin, 2011). The coupling between elision and cross-modal activity in this region for TD children suggests that FG is sensitive to phonological information, but that this sensitivity is dependent on factors such as reader fluency. This is consistent with evidence for processing of orthography and phonology in the left FG in TD readers and a failure to do so in RD in studies by Schurz et al. (2010) and Richlan et al. (2010); reviewed by Richlan (2012). It is also consistent with our recent finding that TD children activate left FG during auditory rhyme judgment tasks, in contrast to children with RD (Desroches et al., 2010). This automatic activation of the orthographic area during spoken language processing suggests the left FG is involved in integration of orthographic and phonological information.

Studies examining audiovisual integration for orthography and phonology at small grain sizes have identified planum temporale (PT) and pSTS as cross-modal integration areas (Blau et al., 2008, 2010). Posterior STS, in particular, is often implicated as an audiovisual convergence zone for both speech and reading (Calvert, 2001; Van Atteveldt et al., 2004; Nath et al., 2011). These studies have shown that audiovisual integration is confined to a relatively narrow time window in which the two stimuli are presented near synchronously. Because pSTS sensitivity to cross-modal congruency distinguished between the reading groups, our results suggest that this region additionally integrates audiovisual information at the whole-word level over a wider time window in which words are presented 1000 ms apart. Our results extend studies examining audiovisual integration in adults and children with dyslexia showing decreased effects of cross-modal congruency in STS for near synchronous presentations as compared to typical readers (Blau et al., 2008, 2010).

Numerous studies on audiovisual integration of letters and speech sounds found congruency effects in PT suggesting that they are due to feedback originating in pSTS (see Van Atteveldt et al., 2009). Moreover, we recently demonstrated that sensitivity of PT (but not pSTS) to cross-modal congruency in TD children engaged in a rhyming task is correlated with reading age (McNorgan et al., 2013). The failure to find a correlation between a measure of phonemic awareness and the cross-modal congruency effect in the PT was thus surprising. This apparent inconsistency may be attributable, however, to the interaction between task demand and region. Reading requires mapping from orthography to phonology and involves the blending of sounds. The orthographic intrusions seen with skilled readers in phonological awareness tasks (Stuart, 1990; Castles et al., 2003), in contrast, suggests that the explicit separation of sounds encourages these readers map from phonology to orthography. McNorgan et al. argued that, as part of the phonological loop, PT should be more sensitive to large grain-size representations, as word representations unfold over time, whereas pSTS should be more sensitive to smaller grain-size representations. Because elision requires analysis of words at smaller grain-sizes, these results are consistent with McNorgan et al., and suggest that cross-modal processes during reading engage the pSTS for online small-grain integration and PT for large-grain integration over longer time windows.

Impaired phonological awareness is commonly cited as the source of reading impairment (Ramus, 2003), though numerous studies show that a failure to automatize letter-sound associations greatly contribute to reading failure (Vellutino et al., 2004; Ehri, 2005; Blau et al., 2010). Our finding that elision skill is significantly correlated with cross-modal lexical processing may reconcile both theories. As a measure of phonological awareness at the phonemic-level, elision has been shown to be a strong predictor of reading performance that differentiates good and poor readers (Vloedgraven and Verhoeven, 2007). Vaessen et al. (2009) suggest that decreased performance on phonological awareness and rapid naming may reflect not only phonological processing, but also impaired automatic integration of orthography and phonology in dyslexic children. We found the strongest correlations between elision and cross-modal congruency in the FG, with greater activation for incongruent orthography. Incongruent orthographic representations have been shown to influence behavioral performance on phonemic awareness tasks such as segmentation, but this effect is more pronounced in TD compared to RD readers (Landerl et al., 1996). These behavioral findings are consistent with neuroimaging data showing that children with RD have reduced sensitivity to grapheme-phoneme consistency compared to TD children (Bolger et al., 2008), and that children with RD lack effects of orthographic familiarity and print specificity in the FG (Van Der Mark et al., 2009). Frost et al. (2009) also found that elision performance was associated with increasing specialization of FG for print. This study, however, did not include a cross-modal condition, instead examining conjunctions and disjunctions between unimodal spoken or print conditions. Consequently, their findings provide indirect evidence to suggest that elision is critically related to cross-modal processes underlying reading. Thus our results are consistent with the body of literature showing a cross-modal influence of orthographic knowledge on phonological processing, and importantly extend it by showing that this influence is reflected in elision skill for normal readers, but not those with reading difficulty. The capacity to carry out audiovisual integration during reading should depend on connectivity between regions mediating phonological and orthographic representations. It is reasonable, therefore, to suppose that the group differences we observe between TD and RD children might be driven by differential development of this connectivity, and this remains a subject of future investigations which might examine task-related connectivity within these groups.

It is interesting to note that both TD and RD children spanned a range of elision skill, and these distributions were largely overlapping. Thus, there were RD children who performed comparably at elision but did not integrate cross-modal information in the same way as the TD children. The greater ability to access and manipulate phonemic knowledge in higher-elision RD children did not translate to improved cross-modal mapping, consistent with the finding that orthographic information is less likely to intrude on phonological tasks for RD readers (Landerl et al., 1996). RD interventions often involve extensive phonological awareness training (Hulme et al., 2012; Youman and Mather, 2012), but the emphasis on phonological awareness may be at the expense of learning to map between modalities. If the critical deficit in dyslexia is in the mapping from orthography to phonology, orthographic knowledge will be less likely to facilitate the development of phonological awareness, and the two skills will be decoupled. This would be reflected by an overall improvement in phonological awareness without a corresponding improvement in cross-modal integration in RD children undergoing phonological awareness training. In a quantitative meta-analysis, Bus and Van Ijzendoorn (1999) showed that phonological awareness training is most effective when paired with alphabetic training (i.e., learning to associate individual letters and corresponding phonemes) suggesting that intervention should focus on the cross-modal mapping between orthographic and phonological representations.

## Conclusion

Our findings suggest that phonemic-level awareness, as measured by elision, arises from increased orthographic fluency (Morais et al., 1979; Ziegler and Goswami, 2005). Thus, elision not only measures phonemic skill and therefore is not a pure measure of meta-phonological skill. Rather, it is a composite skill that taps both phonemic awareness and cross-modal integration. Elision may thus depend on the degree to which cross-modal integration in the FG and pSTS influences phonemic awareness, and a breakdown of this relationship may be indicative of reading difficulty. Behavioral literature examining the changing role of phonological awareness during literacy acquisition shows that it is not an especially strong predictor of reading outcomes in beginning readers (Schatschneider et al., 2004), and that there is a developmental increase in the ability of phonological awareness to predict reading skills (Anthony et al., 2007). Elision may thus not be a strong predictor of phonological processing in pre- or early readers, but rather a marker of facility with integrating orthography and phonology following formal reading instruction.

### Conflict of interest statement

The authors declare that the research was conducted in the absence of any commercial or financial relationships that could be construed as a potential conflict of interest.
